# Effectiveness of Acupuncture for Lateral Epicondylitis: A Systematic Review and Meta-Analysis of Randomized Controlled Trials

**DOI:** 10.1155/2020/8506591

**Published:** 2020-03-20

**Authors:** Yumei Zhou, Yuebao Guo, Rui Zhou, Ping Wu, Fanrong Liang, Zhuoxin Yang

**Affiliations:** ^1^The Fourth Clinical Medical College of Guangzhou University of Chinese Medicine, Shenzhen, GuangDong 518033, China; ^2^College of Acupuncture and Moxibustion, Guangzhou University of Chinese Medicine, Guangzhou, Guangdong 510006, China; ^3^College of Acupuncture and Moxibustion and Tuina, Chengdu University of Traditional Chinese Medicine, Chengdu, Sichuan 610075, China

## Abstract

**Objective:**

This study aimed at assessing the clinical effectiveness of acupuncture for lateral epicondylitis (LE).

**Methods:**

The following databases were systematically searched: China National Knowledge Infrastructure, Chinese Science and Technology Periodical Database, Wan Fang database, Chinese Biomedicine Literature, PubMed, EMBASE, and Cochrane Library from inception to May 2019. Randomized controlled trials (RCTs) meeting the inclusion criteria were included. RevMan 5.3 software was used to conduct meta-analyses. The study quality was evaluated with the Cochrane risk of bias.

**Results:**

Ten RCTs involving 796 individuals were included in this meta-analysis. Three studies reported randomized methods with a specific description. For the analyses of the clinical efficacy rate, acupuncture outperformed sham acupuncture (two RCTs, *n* = 130, *P*=0.15), medicine therapy (two RCTs, *n* = 124, *P*=0.02), and blocking therapy (four RCTs, *n* = 427, *P*=0.0001). For the analyses of the visual analog scale, acupuncture outperformed sham acupuncture (two RCTs, *n* = 92, *P*=0.18), medicine therapy (two RCTs, *n* = 144, *P* < 0.00001), and blocking therapy (two RCTs, *n* = 132, *P*=0.03). The subgroup analyses comparing acupuncture with sham acupuncture therapy revealed heterogeneities. The follow-up information and adverse reactions were not analyzed because of the insufficient number of studies.

**Conclusions:**

Acupuncture appears to be superior to drug or blocking therapy or sham acupuncture therapy for LE. However, considering the low quality of the available trials, further large-scale RCTs with a low risk of bias are needed in the future.

## 1. Introduction

Lateral epicondylitis (LE), commonly known as tennis elbow, is characterized by pain over the lateral epicondyle of the humerus when using the arms, especially when grasping and lifting heavy objects. It is a common orthopedic disorder with a prevalence of 1%–3% in the general population and 7% in handy workers [1, 2]. It mostly affects persons aged 40–50 years with equal sex distribution [2]. The physiopathology of LE is not fully understood, but it is often considered to be caused by repetitive activities and overuse of the extensor carpi radialis brevis [3, 4]. The condition is generally self-limiting lasting for 6–12 months [1]. The pain and function restriction of the elbow joint seriously influence the functional movements and daily activities of patients.

Most patients with LE are likely to receive therapy during the first 6–24 months. Several conservative treatment strategies, such as steroid injections, nonsteroidal anti-inflammatory drugs (NSAIDs), and physical therapies [5], have been used for pain relief. However, the treatment effects and safety of these methods seem to be controversial. Some studies found that steroid injections might induce a strong initial effect but have poor long-term outcomes [6]. Additionally, NSAIDs have long been used for pain alleviation, including oral administration and external application on painful lesions. Previous studies found that NSAIDs might have a better medication route and show bigger treatment effect sizes [7]. However, such treatments are often reported to increase the risk of adverse events, such as fatigue, sleep disturbance, and gastrointestinal intolerance.

As an important part of complementary and alternative therapy, acupuncture is commonly used for a wide range of disorders, such as low back pain, migraine, neck pain, and sciatica. One randomized controlled trial (RCT) [8] found that acupuncture might be related to the long-term reduction in migraine recurrence in 16 weeks after randomization compared with the sham acupuncture and waiting-list groups. Acupuncture was also found to lower the incidences of side effects compared with analgesics [9]. A Cochrane review [10] conducted by Green et al. found that acupuncture was effective in improving the short-term pain of the lateral elbow. However, the results could not be combined in meta-analysis owing to the inclusion of only two trials. Another meta-analysis published in 2004 [11] explored the efficacy of acupuncture for LE compared with sham acupuncture, but no comparison with other conventional interventions, such as drugs and steroid injections, was conducted. Recently, more studies on this topic have been published, and the results remain conflicting. Therefore, this systematic review and meta-analysis of RCTs was performed to compare the efficacy of acupuncture with that of other therapies (sham acupuncture, drugs, and steroid injections) in LE.

## 2. Methods and Analysis

### 2.1. Data Sources and Search Strategy

This meta-analysis was performed in adherence to the Preferred Reporting Items for Systematic Reviews and Meta-Analyses statement [12]. The following electronic databases were searched from inception to May 2019, with no limitation on language: PubMed, EMBASE, Cochrane Library, Chinese Biomedicine Literature (CBM), China National Knowledge Infrastructure (CNKI), Chinese Science and Technology Periodical Database (VIP), and the Wan Fang database. Additionally, a hand search was conducted among the relevant references cited in the selected studies in case some studies were missed by the electronic search.

The search strategy is shown in Table 1 considering PubMed as an example, which was also suitable for other electronic databases.

### 2.2. Eligibility Criteria

Two reviewers (ZYM and GYB) evaluated all identified studies independently according to the following inclusion criteria: (1) study design: RCTs, (2) population: patients diagnosed with LE, (3) intervention: the intervention in the observation group limited to acupuncture therapy (only manual acupuncture or electroacupuncture), and (4) comparison: sham acupuncture, drug, or blocking therapy in the control group.

The exclusion criteria were as follows: (1) nonrandomized control trial (review, meta-analysis, case report, conference abstract, and observational study), (2) duplications, (3) full text unavailable, (4) the control group of studies containing any forms of acupuncture therapy, and (5) the observation group of studies including other therapies except acupuncture, such as medicine.

### 2.3. Data Extraction and Management

Two authors (ZYM and GYB) independently extracted the following information from each study: first author, publication year, sample size, patients (demographic details), interventions, outcome measures, follow-up, reasons for discontinuation, and adverse events. When the data were unclear, attempts were made to contact the corresponding authors. Any disagreements or doubts were figured out by discussion or by consulting another author (WP). Ethical approval was not needed because the data used in this systematic review were not individual patient data and the study had no privacy issues to address.

### 2.4. Quality Assessment

Two investigators (ZYM and GYB) independently assessed the methodological quality of all the included studies. According to the Cochrane Handbook for Systematic Reviews of Interventions, the risk of bias was evaluated in the following items: (1) random sequence generation, (2) allocation concealment, (3) blinding of participants and personnel and blinding of outcome assessment, (4) incomplete outcome data, (5) selective reporting, and (6) other sources of bias. Additionally, the overall evidence levels of the primary and secondary outcomes of acupuncture for LE were assessed using the Grading of Recommendations, Assessment, Development, and Evaluation (GRADE) system. Any disagreements or doubts were figured out by discussion or by consulting another author (WP).

### 2.5. Statistical Analysis

Various outcome measures were used to assess LE, of which the most commonly and frequently adopted measures were selected to extract data for analysis. All analyses were performed with RevMan 5.3 software. The continuous data were presented as mean differences (MD) with 95% confidence intervals (CIs), while dichotomous variables were expressed as a rate ratio (RR) with 95% CIs. The *I*^2^ test was used to address the heterogeneity of the data. *I*^2^ > 50% meant that heterogeneity existed, and the random-effects model was applied for data analysis; otherwise, the fixed-effects model was used. When *I*^2^ was >50%, sensitivity analyses were conducted to explore the source of heterogeneity if the number of identified studies was relative enough (more than three at least). Additionally, forest plots were used to analyze the pooled effect size and individual study effect sizes according to the control interventions (sham acupuncture, drugs, or blocking therapy).

In all analyses, *P* values less than 0.05 were considered statistically significant.

## 3. Results

### 3.1. Results of Literature Retrieval

**Figure 1:**
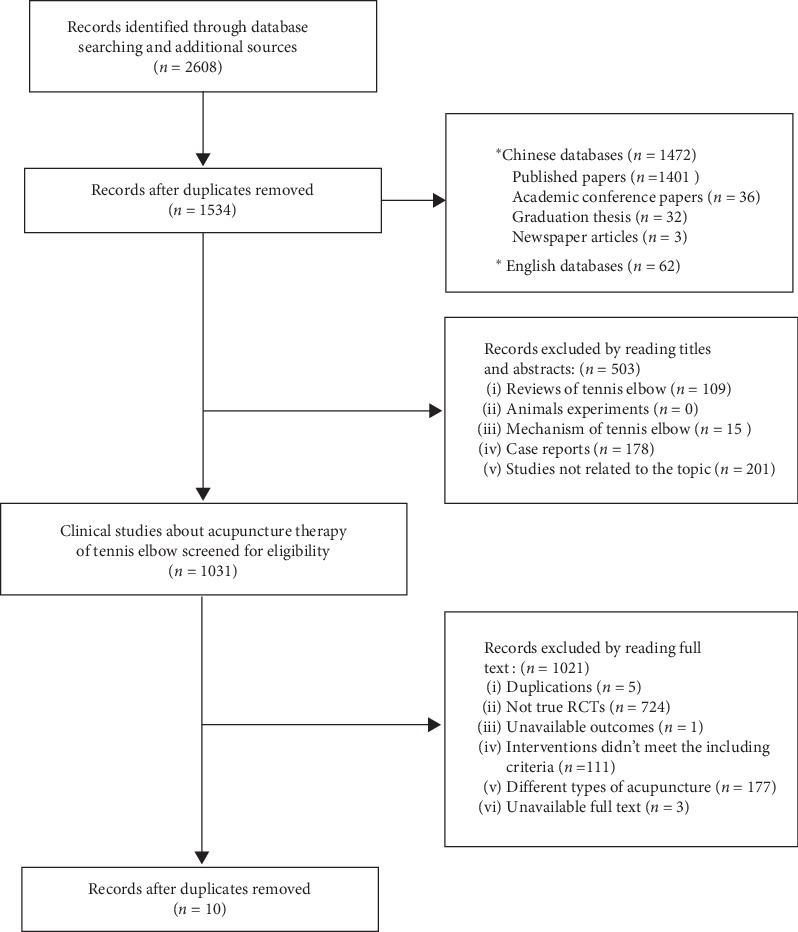
Flowchart of the trial selection process for this systematic review.

A flowchart of search selection and results is shown in Figure 1. A total of 2608 studies were identified by the research strategy. Further, 1074 records were excluded owing to duplicates, and 503 additional records were excluded after reading their titles and abstracts for reasons such as reviews, case reports, mechanistic study of LE, or not related to the topic. After full-text studies were assessed for eligibility, 1021 records were excluded for reasons such as not being an RCT, duplications, irrelevance of the specified patient, intervention, comparison, and outcome, or unavailable full text. Finally, 10 eligible RCTs were included in the meta-analysis.

### 3.2. Characteristics of Included Trials and Literature Search Findings

The 10 RCTs included 796 individual patients in total, of which 431 patients were in the observation group and 365 in the control group. The included 10 studies were published from 1990 to 2018 (median 2001). The sample sizes ranged from 22 to 147 (median 30; interquartile range (IQR) 25–41). All participants met the diagnostic criteria. The mean age ranged from 36.7 to 52.5 years (median 44.56), and all were adults (age ≥ 18 years). The mean course of disease of participants in two studies was less than 1 month [13, 14], and one did not report the course of the disease [15]. For other studies, the mean course of the disease ranged from 4.54 to 7.66 months. All trials included 3–10 treatment sessions (median 10; IQR 6–10). The outcome measures included the clinical efficacy rate, visual analog scale (VAS), and functional recovery-related scales. The clinical efficacy rate was reported in eight trials, and VAS were evaluated in six trials. The detailed characteristics of the included studies are listed in Table 2.

### 3.3. Risk of Bias in Included RCTs

**Figure 2:**
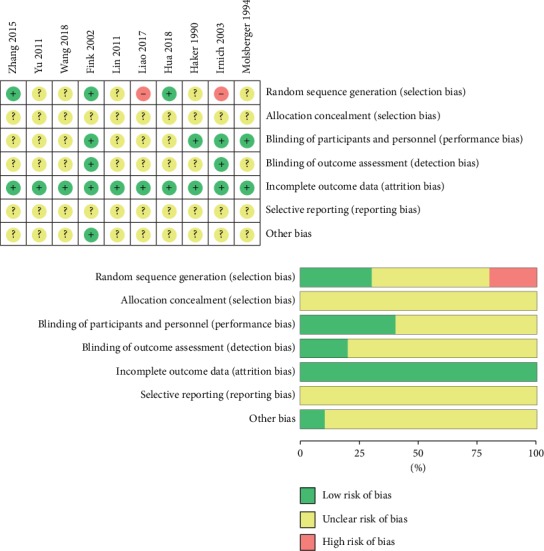
Plots of risk of bias.

The plots of the risk of bias and methodological quality of the included studies are shown and summarized in Figure 2. All of the included RCTs used randomization; however, only three studies were randomized by random number tables [13, 17, 20] with a low risk of bias. Another two studies were randomized by the registration order [16, 19] and considered to have a high risk of bias. The details of allocation concealment were unclear in all included studies. Four studies were reported with blinding [15–18]. Both evaluators and patients in the study were blinded in two RCTs [16, 17], and the participants were blinded in two RCTs [15, 18]. Whether the blinding methods in the remaining six RCTs were performed was unclear. Additionally, two RCTs [17, 18] reported patient dropouts. Selective reporting and other biases were unclear in all included studies.

Furthermore, the overall evidence level of meta-analyzable outcome measures was rated “low” (50%, 1/2) or “moderate” (50%, 1/2) by the GRADE approach (Table 3).

### 3.4. Outcome Measures

A total of 10 RCTs were included; the clinical efficacy rate was reported in 8 studies [13–15, 18–22], while VAS was reported in 6 studies [13, 16, 17, 20–22]. Other outcome measures were not used for analysis because of an insufficient number of studies. Additionally, the follow-up results were also not analyzed owing to the various time points.

#### 3.4.1. Clinical Efficacy Rate

**Figure 3:**
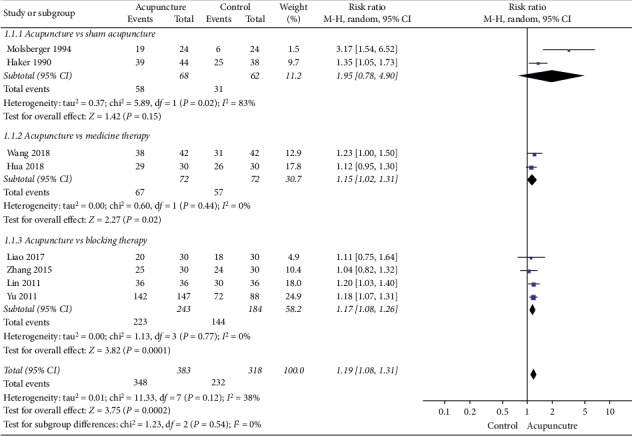
Forest plot showing the clinical efficacy rate of acupuncture treatment versus sham acupuncture therapy treatment, versus blocking therapy, and versus medicine therapy for lateral epicondylitis.

The eight studies [13–15, 18–22] with the assessments of clinical efficacy rate were divided into three parts to perform the meta-analysis according to the different types of comparison groups (Figure 3).

1. Acupuncture versus sham acupuncture: the meta-analysis of two RCTs [15, 18] showed significant heterogeneity (*χ*^2^ = 5.89; *P*=0.02; *I*^2^ = 83%). The combined results showed that the clinical efficacy rate improved in acupuncture therapy (observation group) compared with the sham acupuncture therapy (control group) (RR = 1.95; 95% CI: 0.78–4.90; *P*=0.15).
2. Acupuncture versus medicine therapy: the two studies [13, 22] showed homogeneity (*χ*^2^ = 0.60; *P*=0.44; *I*^2^ = 0%). The pooled results showed that the clinical efficacy rate improved significantly in acupuncture therapy (observation group) compared with the medicine therapy (control group) (RR = 1.15; 95% CI: 1.02–1.31; *P*=0.02).
3. Acupuncture versus blocking therapy: the meta-analysis of these four RCTs [14, 19–21] showed homogeneity in the consistency of the trial results (*χ*^2^ = 1.13; *P*=0.77; *I*^2^ = 0%). The combined results showed that the clinical efficacy rate improved significantly in acupuncture therapy (observation group) compared with the blocking therapy (control group) (RR = 1.17; 95% CI: 1.08–1.26; *P*=0.0001).

#### 3.4.2. Visual Analog Score

**Figure 4:**
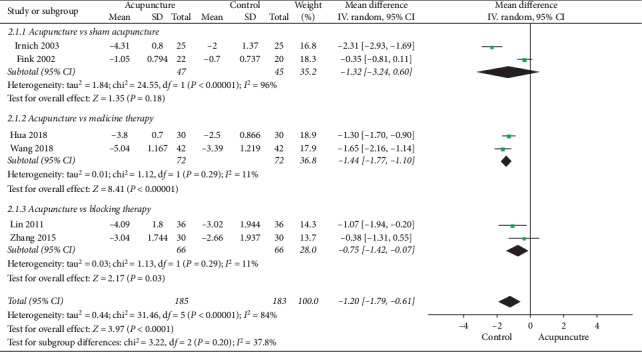
Forest plot showing a VAS score of acupuncture treatment versus sham acupuncture therapy treatment, versus blocking therapy, and versus medicine therapy for lateral epicondylitis.

The six studies [13, 16, 17, 20–22] involving VAS assessments were divided into three parts to perform the meta-analysis according to the different types of comparison groups (Figure 4):

1. Acupuncture versus sham acupuncture: the two studies [16, 17] showed significant heterogeneity (*χ*^2^ = 24.55; *P* < 0.00001; *I*^2^ = 96%), and the MD was −1.32 (95% CI: −3.24 to 0.60).
2. Acupuncture versus medicine therapy: the two studies [13, 22] showed no heterogeneity (*χ*^2^ = 1.12; *P*=0.29; *I*^2^ = 11%). The pooled results indicated that acupuncture could decrease the VAS score more significantly compared with medicine therapy (MD = –1.44; 95% CI: −1.77 to −1.10; *P* < 0.00001).
3. Acupuncture versus blocking therapy: the two studies [20, 21] showed no heterogeneity (*χ*^2^ = 1.13; *P*=0.29; *I*^2^ = 11%). The pooled results indicated that acupuncture could decrease the VAS score more significantly compared with blocking therapy (MD = −0.75; 95% CI: −1.42 to −0.07; *P*=0.03).

## 4. Discussion

### 4.1. Evaluation of the Methodology of the Trials

Acupuncture therapy has been considered to be an effective and feasible intervention for LE. In this systematic review and meta-analysis, 10 RCTs, with a total of 796 participants, were included to evaluate the effect of acupuncture on LE. Randomization and allocation concealment of the 10 RCTs were not significantly reported, which might have led to a high risk of selection bias. In addition, in these 10 RCTs, only 4 studies were reported with blinding of evaluators and/or participants, which might have also resulted in a considerable risk of bias. Moreover, 6 out of 10 studies were conducted in China and published in Chinese, and these 6 studies did not report the randomization and blinding method, resulting in publication bias. The quality and authenticity of the RCT methods were quite difficult to judge. Therefore, the number of high-quality RCTs was inadequate to provide powerful evidence.

### 4.2. Summary of Therapy Efficacy

In the present study, the meta-analysis results indicated that acupuncture could exert a higher total effective response rate and was superior in decreasing the VAS score compared with other treatment interventions (sham acupuncture, medicine therapy, and blocking therapy). A few side effects were reported, for instance, a participant dropout because of pricking pain resulting from needling [17], which were insufficient for quantitative analysis.

A previous meta-analysis on the same topic was conducted and published by the Cochrane Library in 2002 [10]. In detail, the Cochrane study included 4 RCTs with 281 participants, of which 2 studies [15, 18] met the inclusion criteria and were also included in the present study. In this Cochrane study, no reliable conclusions could be drawn on the effect of acupuncture on tennis elbow because of the poor quality and inadequate numbers of the included trials.

Another three meta-analyses [23–25] were conducted to understand the effects of laser acupuncture therapy on tennis elbow, which were designed mainly to compare the efficacy of laser therapy against nonlaser or placebo laser therapy with zero output. Laser acupuncture therapy was physiotherapy with irradiation emitting on acupoints, tender points, or myofascial trigger points, rather than inserting needles into the body and not achieving a needling sensation of *Deqi*. Therefore, it is believed that laser acupuncture therapy is not equivalent to conventional acupuncture therapy. Hence, the trials with laser acupuncture intervention were not included in this study.

In the present meta-analysis, RCTs were found by searching in the English databases PubMed, EMBASE, and the Cochrane Central Register of Controlled Trials, and the Chinese databases VIP, Wan Fang, CNKI, and CBM from inception to May 2019. The main findings were that acupuncture or electropuncture therapy could obviously improve the clinical efficacy rate and significantly decrease the VAS score compared with sham acupuncture, blocking therapy, and drug therapies (control groups). The result was in line with the findings of a previous study [11], showing that acupuncture could successfully help manage pain and effectively alleviate tennis elbow.

In the subgroup analysis of the sham-controlled studies, the combined results found greater efficacy in the verum acupuncture group than in the sham acupuncture group. However, heterogeneity still existed. Only acupuncture (or electropuncture) therapy was required as the intervention method without any other therapies in the observation group, so as to decrease the interference. However, the acupuncture operations administered, such as acupoint selection, acupuncture stimulation intensity, duration, frequency, and total number of treatment sessions, were different, which might be the source of heterogeneity. Additionally, the style of nonacupoint selection and treatment protocol in the sham acupuncture group would also induce the heterogeneity (see Table 2 for detailed information).

Additionally, among the 10 RCTs included in this study, a subgroup with 4 RCTs [14, 19–21] used the steroid for local injections (blocking therapy), and two studies [13, 22] in another subgroup used drug therapy. The local steroid injections and NSAIDs were commonly used for treating LE in the clinic [26]. The two intervention methods have been confirmed to be effective in relieving acute pain [27–29], but have no obvious superiority regarding the long-term analgesic effect and may also induce some side effects [28, 30]. In the present study, the clinical efficacy response rates of acupuncture treatment (observation group) were 1.17 times and 1.15 times more than that of the blocking therapy and drug therapy, respectively. Moreover, VAS was commonly used to assess the degree of pain. In the subgroup analyses, the pooled analysis results also indicated that acupuncture could decrease the VAS score more significantly. Unfortunately, no follow-up was conducted in most studies or the time points of follow-up varied; hence, the long-time efficacy of acupuncture could not be compared with that of blocking therapy and drug therapy.

Furthermore, in addition to pain assessments, other objective outcome measures, such as the recovery of function and returning to work, were also important variables for LE. However, only three trials [17, 20, 22] reported the information on elbow joint activity with different scales in this study. Owing to the insufficient number of studies, the functional recovery could not be assessed comprehensibly, which was in line with the previous meta-analysis.

### 4.3. Limitations

This meta-analysis had several potential limitations that should be taken into account. First, most included studies were of poor methodological quality, especially missing the details of blinding and randomization procedures, leading to a high risk of bias. Second, the study included only 10 trials, and the publication numbers in each subgroup were few. Additionally, the sample sizes of most trials were small. Third, the acupuncture protocols of the included trials varied, such as acupoint selection, depth of insertion, and total number of treatments. The interventions might have been the source of heterogeneity. Finally, the outcome measures were not consistent across studies, especially in the assessments for the recovery of function; hence, the studies could not be combined for meta-analysis. Additionally, the side effects or complications of acupuncture were reported so insufficiently that other meaningful clinical endpoints could not be evaluated.

More stringent RCTs with large sample sizes are needed to be designed with randomization, allocation concealment, and blinding. Further, the issues of acupuncture treatment protocols and more objective outcomes should be addressed. Meanwhile, further studies should focus on not only the efficacy but also the safety of acupuncture.

## 5. Conclusions

In conclusion, despite the limitations of the methodology, the results of this meta-analysis indicated that acupuncture therapy might be more effective than drugs and blocking therapy in improving the clinical efficacy rate and decreasing the VAS score. Well-designed RCTs with larger sample sizes and long-term follow-up on these topics are still needed to supply reliable evidence on the efficacy of acupuncture for treating LE.

## Figures and Tables

**Table 1:** Search strategy of acupuncture for lateral epicondylitis in the PubMed database.

No.	Keywords
(1)	Randomized controlled trial
(2)	Controlled clinical trials
(3)	Randomly
(4)	Randomized
(5)	Trial
(6)	Placebo
(7)	1 or 2–6
(8)	Lateral humeral epicondylitis
(9)	Lateral epicondylitis
(10)	Tennis elbow
(11)	8 or 9-10
(12)	Acupuncture
(13)	Acupuncture therapy
(14)	Acupoints
(15)	Body acupuncture
(16)	Scalp acupuncture
(17)	Electroacupuncture
(18)	Fire needle
(19)	Plum-blossom needle
(20)	Elongated needle
(21)	Intradermal needle
(22)	12 or 13–21
(23)	7 and 11 and 22

**Table 2:** Characteristics of the included studies.

First author	Sample size (observation/control)	Dropout rate	Intervention (in the observation group)	Intervention (in the control group)	Course of treatment	The main outcomes
Irnich et al. [16]	50 (25/25)	None	Verum acupuncture: LI 4, LI 10, SJ 5, SI 3, GB 34	Sham acupuncture: points: one thumb, with away from those used in the observation group	3 treatments within 10 days	Pressure pain threshold (PPT) Pain-free grip strength (GS) NRS (same to VAS, assessment on pain on 0–10 scale) All assessments after treatments and 14-day follow-up

Fink et al. [17]	45 (23/22)	3 at 2-week and 2 more at 2-month follow-up	Verum acupuncture: LI 10, LI 11, Lu 5, LI 4, SJ 5, one A-Shi point	Sham acupuncture: points: 5 cm away from the points used in the observation group	10 treatments 2 times/week within 5 weeks	Pain reduction percentage VAS (pain assessed at rest, in motion, during exertion, and frequency on 0–5 scale) Functional impairment assessed with DASH questionnaire All assessments after treatments and 2-month follow-up

Molsberger and Hille [15]	48 (24/24)	None	Verum acupuncture: GB 34 (on ipsilateral leg)	Sham acupuncture: (stimulation with pencil-like probe to simulate needle insertion) Acupuncture point UB 13	1 treatment	Clinical efficacy rate VAS (pain assessed on 0–10 scale) Pain relief score

Haker and Lundeberg [18]	82 (44/38)	4 after 10th treatment Another 5 at 3 months	Verum acupuncture: LI 10, LI 11, LI 12, Lu 5, SJ 10	Sham acupuncture: same acupoints but superficial needle insertion	10 treatments in all 2-3 times/week	Clinical efficacy rate, the vigorimeter test Assessments after treatments, at 3-month and 1-year follow-up

Liao Leshan [19]	60 (30/30)	None	Acupuncture therapy: LI10, SJ 5, LI 4, LI 12 (affected side), once a day, 2 weeks	Blocking therapy: local injection of 0.5 ml triamcinolone acetate A injection plus 3 ml lidocaine, once a week, 2 weeks	10 treatments in the observation group 3 treatments in the control group	Clinical efficacy rate
Zhang Xiaoyang and Huang [20]	60 (30/30)	None	Acupuncture therapy: LI11, LI10, LI13, LI 1, Ah-Shi, LI3 once every other day, 2 weeks	Blocking therapy: local injection of 1% 4 ml lidocaine injection and 1 ml prednisolone at tenderness point and LI11 once for ten days, 2 weeks	10 treatments in the observation group 2 treatments in the control group	Clinical efficacy rate, VAS (pain assessed on 0–10 scale) Elbow joint activity score (rotation function assessed on 0–8 scale) All assessments after first therapy, all treatments and at 1-month follow-up

Min [21]	72 (36/36)	None	Acupuncture therapy: points: the most tenderness point, three points around the tenderness points, and LI11. Once every other day, 2 weeks	Blocking therapy: local injection of 2% 1.5 ml procaine injection and 5 ml prednisolone suspension at tenderness point once for ten days, 2 weeks	10 treatments in the observation group 2 treatments in the control group	Clinical efficacy rate VAS (pain assessed on 0–10 scale) Both assessments after treatments and 2-month follow-up

Hongrui [14]	235 (147/88)	None	Acupuncture therapy: LI4, LI7, LI9, LI10 (affected side) once a day, 3 times a week, 3 weeks	Blocking therapy: local injection of 1% 4 ml lidocaine injection and 50 mg prednisolone at tenderness point, once a week, 3 weeks	9 treatments in the observation group 3 treatments in the control group	Clinical efficacy rate

Yuanli [22]	84 (42/42)	None	Electroacupuncture therapy: cervical Jiaji 5–7 (EX-B2, affected side), SI 11, A-Shi points, LI 11, LI 10, SJ 5 once a day, 5 times a week, 2 weeks	Drug therapy group: oral meloxicam tablets 7.5 mg once a day for 2 weeks	10 treatments in the observation group 14 treatments in the drug group	Clinical efficacy rate VAS (pain assessed on 0–10 scale) Elbow function score scale (function assessed on 0–100 scale)

Hui [13]	60 (30/30)	None	Acupuncture therapy: points: 4 points at 0.5 cm away from the tenderness point at 3, 6, 9, 12 o'clock, once every other day, 2 weeks	Drug therapy group: oral celecoxib capsules 200 mg and external application of votalin ointment twice a day, 2 weeks	7 times in the observation group 28 times in the drug group	Clinical efficacy rate VAS (pain assessed on 0–10 scale) Both assessments after treatments and 3-month follow-up

**Table 3:** GRADE analyses: acupuncture treatment for lateral epicondylitis.

Primary and secondary outcomes	No. of study (subjects)	Risk of bias	Inconsistence	Indirectness	Imprecision	Publication bias	Overall quality of evidence^a^
Clinical efficacy rate	8 (701)	No	Serious^b^	No	No	No	+/+/+/−/; moderate
VAS	6 (368)	No	Serious^b^	No	Serious^c^	No	+/+/−/−/; low

GRADE = Grading of Recommendations Assessment, Development, and Evaluation; VAS = visual analogue scale. ^a^GRADE working group grades of evidence: high quality = further research is very unlikely to change our confidence in the estimate of effect; moderate quality = further research is likely to have an important impact on our confidence in the estimate of effect and may change the estimate; low quality = further research is very likely to have an important impact on our confidence in the estimate of effect and is likely to change the estimate; very low quality = we are very uncertain about the estimate. ^b^Meta-analytic results presented a serious inconsistency when *I*^2^ values were greater than 20% in the *Q* statistics. ^C^Meta-analytic results presented a serious imprecision when 95% CI = effect size in the *Q* statistics.
