# Corrigendum to “Effectiveness of Acupuncture for Lateral Epicondylitis: A Systematic Review and Meta-Analysis of Randomized Controlled Trials”

**DOI:** 10.1155/2022/9764940

**Published:** 2022-02-17

**Authors:** Yumei Zhou, Yuebao Guo, Rui Zhou, Ping Wu, Fanrong Liang, Zhuoxin Yang

**Affiliations:** ^1^The Fourth Clinical Medical College of Guangzhou University of Chinese Medicine, Shenzhen, Guangdong 518033, China; ^2^College of Acupuncture and Moxibustion, Guangzhou University of Chinese Medicine, Guangzhou, Guangdong 510006, China; ^3^College of Acupuncture and Moxibustion and Tuina, Chengdu University of Traditional Chinese Medicine, Chengdu, Sichuan 610075, China

In the article titled “Effectiveness of Acupuncture for Lateral Epicondylitis: A Systematic Review and Meta-Analysis of Randomized Controlled Trials” [1], some of the references in Table 2 were formatted incorrectly. Corrected Table 2 and the references are as follows:

  [13] H. Hua, “Clinical observation on the treatment of external humeral epicondylitis by hysteresis acupuncture combined with acupuncture manipulation,” *Journal of New Chinese Medicine*, vol. 50, no. 11, pp. 196–198, 2018.
  [14] H. R. Yu, “Therapeutic effect of acupuncture on 147 cases of external humeral epicondylitis,” *Hebei Journal of Traditional Chinese Medicine*, vol. 33, no. 6, pp. 890–891, 2011.
  [19] L. S. Liao and W. J. Guo, “Ironing combined with acupuncture therapy for 30 cases of tennis elbow,” *Journal of External Therapy of TCM*, vol. 26, no. 6, pp. 10–11, 2017.
  [20] X. Y. Zhang, Q. Liu and M. Huang, “Effect observation of 30 cases of tennis elbow treated by fire needle,” *Journal of Sichuan of Traditional Chinese Medicine*, vol. 33, no. 4, pp. 168–170, 2015.
  [21] M. Lin, “Effect observation of 36 cases of external humerus epicondylitis treated with elbow five needle,” *Shandong Journal of Traditional Chinese Medicine*, vol. 30, no. 9, pp. 639–640, 2011.
  [22] Y. L. Wang, “Clinical observation of electroacupuncture in treatment of refractory external humeral epicondylitis,” *AsiaPacific Traditional Medicine*, vol. 14, no. 8, pp. 165–166, 2018.

## Figures and Tables

**Table 2:** Characteristics of the included studies.

First author	Sample size (observation/control)	Dropout rate	Intervention (in observation group)	Intervention (in control group)	Course of treatment	The main outcomes
Irnich [16]	50 (25/25)	None	Verum acupuncture: LI 4, LI 10, SJ 5, SI 3, GB 34	Sham acupuncture; points: one thumb-with away from those used in observation group	3 treatments within 10 days	Pressure pain threshold (PPT), pain-free grip strength (GS), NRS (same to VAS, assessment on pain on 0–10 scale), all assessments after treatments and 14-day follow-up
Fink [17]	45 (23/22)	3 at 2-week follow-up and 2 more at 2-month follow-up	Verum acupuncture: LI 10, LI 11, Lu 5, LI 4, SJ 5, one Ah-Shi point	Sham acupuncture; points: 5 cm away from the points used in observation group	10 treatments for 2 times/week within 5 weeks	Pain reduction percentage, VAS (pain assessed at rest, in motion, during exertion and frequency on 0–5 scale) functional impairment assessed with DASH questionnaire, all assessments after treatments and 2-month follow-up
Molsberge [15]	48 (24/24)	None	Verum acupuncture: GB 34 (on ipsilateral leg)	Sham acupuncture: (stimulation with pencil-like probe to simulate needle insertion) acupuncture point UB 13	1 treatment	Clinical efficacy rate, VAS (pain assessed on 0–10 scale) pain relief score
Haker [18]	82 (44/38)	4 after 10th treatment, another 5 at 3 months	Verum acupuncture: LI 10, LI 11, LI 12, Lu 5, SJ 10	Sham acupuncture: same acupoints but superficial needle insertion	10 treatments for all 2-3 times/week	Clinical efficacy rate, the Vigorimeter test assessments after treatments at 3-month and 1-year follow-up
Liao [19]	60 (30/30)	None	Acupuncture therapy: LI10, SJ 5, LI 4, LI 12 (affected side); once a day, 2 weeks	Blocking therapy: local injection of 0.5 ml triamcinolone acetate A injection plus 3 ml lidocaine, once a week, 2 weeks	10 treatments in observation group; 3 treatments in control group	Clinical efficacy rate
Zhang [20]	60 (30/30)	None	Acupuncture therapy: LI11, LI10, LI13, LI 1, Ah-Shi, I3; once every other day, 2 weeks	Blocking therapy: local injection of 1% lidocaine injection 4 ml and prednisolone 1 ml at tenderness point and LI11; once for ten days, 2 weeks	10 treatments in observation group; 2 treatments in control group	Clinical efficacy rate, VAS (pain assessed on 0–10 scale), elbow joint activity score (rotation function assessed on 0–8 scale), all assessments after first therapy, after all treatments, and at 1-month follow-up
Lin [21]	72 (36/36)	None	Acupuncture therapy; points: the most tenderness point, three points around the tenderness points, and LI11; once every other day, 2 weeks	Blocking therapy: local injection of 2% procaine injection 1.5 ml and prednisolone suspension 5 ml at tenderness point; once for ten days, 2 weeks	10 treatments in observation group; 2 treatments in control group	Clinical efficacy rate, VAS (pain assessed on 0–10 scale), both assessments after treatments and 2-month follow-up
Yu [14]	235 (147/88)	None	Acupuncture therapy: LI4, LI7, LI9, LI10 (affected side); once a day, 3 times a week, 3 weeks	Blocking therapy: local injection of 1% lidocaine injection 4 ml and prednisolone 50 mg at tenderness point; once a week, 3 weeks	9 treatments in observation group; 3 treatments in control group	Clinical efficacy rate
Wang [22]	84 (42/42)	None	Electroacupuncture therapy; cervical Jiaji 5–7 (EX-B2, affected side), SI 11, Ah-Shi points, LI 11, LI 10, SJ 5; once a day, 5 times a week, 2 weeks	Drug therapy group: oral meloxicam tablets 7.5 mg, once a day for 2 weeks	10 treatments in observation group; 14 treatments in drug group	Clinical efficacy rate, VAS (pain assessed on 0–10 scale), elbow function score scale (function assessed on 0–100 scales)
Hua [13]	60 (30/30)	None	Acupuncture therapy; points: 4 points at 0.5 cm away from the tenderness point at 3, 6, 9, and 12 o'clock; once every other day, 2 weeks	Drug therapy group: oral celecoxib capsules 200 mg and external application of Votalin ointment twice a day, 2 weeks	7 times in observation group; 28 times in drug group	Clinical efficacy rate, VAS (pain assessed on 0–10 scale), both assessments after treatments and 3-month follow-up
